# Targeted Therapy for Antihistamine-Refractory Chronic Spontaneous Urticaria: A Critical Narrative Review and an Evidence-Graded Approach to Agent Selection

**DOI:** 10.3390/antib15050088

**Published:** 2026-09-17

**Authors:** Luca Guarino, Giuseppe Rizzuto, Vincenzo Coppolelli, Alessia Scipione, Mirella Scattone, Luca Gargano, Pietro Scribani Rossi, Ester Del Duca, Annunziata Dattola, Giovanni Pellacani, Steven Nisticò, Camilla Chello

**Affiliations:** Section of Dermatology, Department of Clinical Internal, Anesthesiological and Cardiovascular Sciences, Sapienza University of Rome, 00161 Rome, Italy; giuseppe.rizzuto@uniroma1.it (G.R.); vincenzo.coppolelli@uniroma1.it (V.C.); alessia.scipione@uniroma1.it (A.S.); mirella.scattone@uniroma1.it (M.S.); lucagargano1995@gmail.com (L.G.); pietro.scribanirossi@uniroma1.it (P.S.R.); ester.delduca@gmail.com (E.D.D.); nancydattola@gmail.com (A.D.); steven.nistico@uniroma1.it (S.N.); camilla.chello@gmail.com (C.C.)

**Keywords:** chronic spontaneous urticaria, antibody-based therapy, omalizumab, dupilumab, remibrutinib, barzolvolimab, BTK inhibitor, endotype, treatment selection, precision medicine

## Abstract

**Background/Objectives:** Chronic spontaneous urticaria (CSU) is a mast-cell-driven disease with autoimmune mechanisms and an incompletely clarified aetiology, in which a substantial proportion of patients remain symptomatic despite second-generation H1-antihistamines; until recently, omalizumab was effectively the only targeted option, leaving a considerable non-responder fraction. Between February 2024 and September 2025 the landscape changed: dupilumab (anti-IL-4Rα) and the first oral Bruton tyrosine kinase (BTK) inhibitor, remibrutinib, were approved for CSU; omalizumab biosimilars reached the market; the higher-affinity anti-IgE antibody ligelizumab was discontinued; and the investigational anti-KIT antibody barzolvolimab advanced to phase 3—creating new therapeutic opportunities for non-responders while leaving the choice among these second-line options unresolved by current guidelines. We provide a critical synthesis and propose an evidence-graded process for agent selection, together with a proposed repositioning of the available agents, taking the 2022 EAACI/GA^2^LEN/EuroGuiDerm/APAAACI guideline as the acknowledged reference. **Methods:** Critical narrative review with a structured but non-systematic search (PubMed, ClinicalTrials.gov) and a pre-specified evidence hierarchy (guideline to expert opinion); each clinical claim is annotated by its level of evidence. **Results:** Four agents are appraised in turn—omalizumab (anti-IgE, first-line), dupilumab (anti-IL-4Rα), remibrutinib (oral BTK inhibitor, small-molecule comparator) and barzolvolimab (anti-KIT, investigational). Current evidence appears to support omalizumab as first-line, with mechanism-guided second-line choices conditioned on endotype, comorbidity, prior biologic exposure and safety. **Conclusions:** The complexity of CSU and the incomplete response to currently available therapies support a patient-tailored approach to treatment selection.

## 1. Introduction

Chronic spontaneous urticaria (CSU) is a mast-cell-driven inflammatory disease defined by recurrent wheals, angioedema or both, persisting for at least six weeks without an identifiable external trigger. The 2020 systematic review and meta-analysis by Fricke and colleagues estimated a global point prevalence of 0.5–1%, with substantial regional variation (Asia 1.4%, 95% CI 0.5–2.9; Europe 0.5%, 0.2–1.0; North America 0.1%, 0.1–0.1) and a female predominance [1]. A meaningful proportion of patients remain symptomatic despite high-dose second-generation H1-antihistamines (sgH1-AH); the disease burden in sleep, productivity and mental health is substantial.

For more than a decade, the therapeutic ladder followed a familiar three-step structure: sgH1-AH, dose-escalated sgH1-AH up to fourfold, and add-on omalizumab. Cyclosporine was reserved for omalizumab failures or for patients without biologic access. The 2022 EAACI/GA^2^LEN/EuroGuiDerm/APAAACI urticaria guideline codified this algorithm and remains the reference document [2].

Why this review is needed now. Within an eighteen-month window (February 2024 to September 2025), three regulatory events and two informative failures collectively reorganised the field. Dupilumab (anti-IL-4Rα) was approved for CSU in Japan (February 2024) and in the United States (April 2025) following the LIBERTY-CSU CUPID programme, in which Study A and Study C met their primary endpoints but Study B did not [3,4,5]. The first omalizumab biosimilar (CT-P39) entered the European market in March 2024 and the US market in March 2025 [4]. Remibrutinib (oral covalent BTK inhibitor) received FDA approval on 30 September 2025 (marketed as Rhapsido) for adults symptomatic on sgH1-AH, on the basis of REMIX-1 and REMIX-2 [6,7]. In parallel, ligelizumab—a higher-affinity successor to omalizumab—did not demonstrate superiority over omalizumab in PEARL-1 and PEARL-2 and was discontinued in CSU [8]. The anti-KIT antibody barzolvolimab entered phase 3 in EMBARQ-CSU1 and EMBARQ-CSU2 with primary completion expected in October 2026, and the second anti-KIT antibody briquilimab entered phase 1b/2a (BEACON) [9]. The first head-to-head trial in this new landscape, RECLAIM (NCT06868212), began recruiting in July 2025 [10].

For the first time in CSU, clinicians face several mechanistically distinct targeted options whose relative positioning the current guideline does not yet resolve. This review takes the 2022 EAACI/GA^2^LEN/EuroGuiDerm/APAAACI guideline as its reference and develops, beyond the guideline ladder, an evidence-graded process for selecting the most appropriate targeted agent for the individual patient, together with a proposed repositioning of the available agents in clinical practice. Each selection step and each repositioning point is annotated with its level of evidence and is framed as a proposal and as hypothesis-generating, not as established or guideline-endorsed practice. The review first appraises each agent critically (Section 4), then compares them (Section 5), and finally integrates the evidence into the selection process (Section 6).

Relationship to existing literature and contribution. The 2022 EAACI/GA^2^LEN/EuroGuiDerm/APAAACI guideline predates the CSU approvals of dupilumab and remibrutinib and the phase 3 development of anti-KIT therapy, and does not resolve how to position mechanistically distinct second-line agents once omalizumab has failed [2]. Recent reviews comprehensively describe this expanding armamentarium [4,11,12], but remain descriptive syntheses organised by agent; to our reading, none operationalises the second-line decision at the level of the individual patient. The contribution of this review is therefore operational: an explicit, evidence-graded selection algorithm anchored to the guideline; a predictive-factor-to-mechanism mapping; a safety- and population-driven rule for the BTK-inhibitor-versus-anti-KIT choice; and a pragmatic surrogate for type IIb autoimmune CSU. Each element is presented as a proposal requiring prospective validation, not as guideline-endorsed practice; we make no claim of primacy.

## 2. Methods

### 2.1. Article Type and Approach

**Figure 1:**
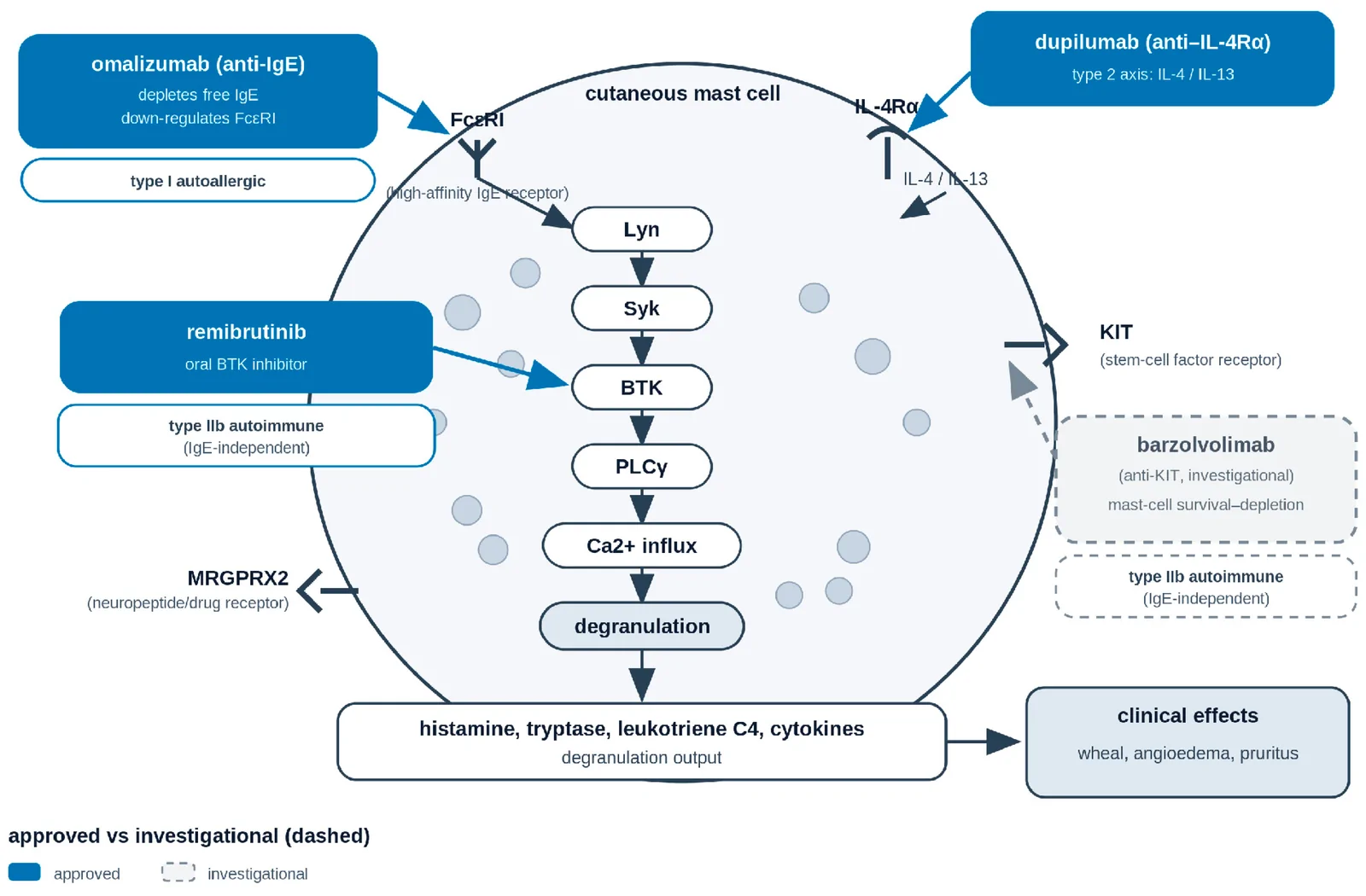
Pathophysiology of CSU and the mechanism of action of the four targeted agents. Schematic of a cutaneous mast cell showing the four activating pathways (FcεRI, IL-4Rα, KIT, MRGPRX2) and the intracellular FcεRI signalling cascade (Lyn, Syk, BTK, PLCγ). Each agent is mapped to the node it targets—omalizumab (anti-IgE) upstream of FcεRI; dupilumab (anti-IL-4Rα) on the type 2 axis; remibrutinib at the intracellular BTK convergence; barzolvolimab (anti-KIT, investigational) at mast-cell survival/depletion—together with the endotype most likely to benefit (type I autoallergic versus type IIb autoimmune).

This article is a critical narrative review with structured methodology. Where indirect comparisons are mentioned, they are taken from a published network meta-analysis [13] and engaged critically. The intent is to integrate, compare, appraise and propose, not to chronologically describe the literature. GPT-5.6 was used to correct Figure 1.

### 2.2. Search Strategy

PubMed and ClinicalTrials.gov were the primary sources, supplemented by the 2022 international guideline, FDA and EMA regulatory communications, and registered phase 3 protocols. Search terms combined the indication (“chronic spontaneous urticaria” OR “chronic urticaria” OR CSU) with drug names and mechanism classes. The literature cut-off is 23 June 2026. A final pre-submission refresh is planned within two weeks of submission (Box 1).

Box 1 **Search strategy**

  Databases: PubMed and ClinicalTrials.gov, supplemented by FDA/EMA regulatory communications and the 2022 international guideline. Representative search strings (combined with Boolean operators and MeSH where available): (“chronic spontaneous urticaria” OR “chronic urticaria” OR CSU) AND (omalizumab OR dupilumab OR remibrutinib OR barzolvolimab OR “anti-KIT” OR “BTK inhibitor”); (CSU) AND (endotype OR “type IIb” OR biomarker * OR predict * OR responder *); (CSU) AND (“total IgE” OR basopenia OR FceRI OR ASST OR “basophil activation test” OR D-dimer OR anti-TPO); agent-specific trials (REMIX, CUPID, PEARL, EMBARQ-CSU, RECLAIM) and safety terms. Literature cut-off 23 June 2026. Evidence hierarchy (pre-specified): G guideline · 1 phase 3 RCT · 2 SR/MA · 3 phase 2 RCT · 4 real-world · 5 mechanistic review · 6 expert/conference. This is a structured but non-systematic search: no PRISMA flow diagram and no pooling of effect sizes were undertaken.

### 2.3. Evidence Hierarchy

References were selected according to: (G) international guideline; (1) phase 3 RCT in a peer-reviewed journal; (2) systematic review and/or meta-analysis; (3) phase 2 RCT; (4) prospective real-world cohort or registry; (5) mechanistic or narrative review; (6) expert opinion or conference abstract. Each recommendation in Section 6 and Section 7 is annotated with the highest level of evidence supporting it. Where the highest level is 5 or 6, the recommendation is explicitly framed as hypothesis-generating.

### 2.4. Endpoint Considerations

The Urticaria Activity Score over 7 days (UAS7) is the canonical endpoint of CSU clinical trials, but it captures a partial picture of disease burden. The Urticaria Control Test (UCT) is more sensitive to control state in routine care; the Angioedema Activity Score (AAS7) and the Angioedema Control Test (AECT) better capture angioedema-predominant phenotypes; the Chronic Urticaria Quality of Life Questionnaire (CU-Q2oL) and the Dermatology Life Quality Index (DLQI) capture patient-reported well-being. Trial-to-trial comparison is complicated by the variable use and pre-specification of these instruments. Where this review reports UAS7 outcomes, the reader should bear in mind that itch, sleep and angioedema dimensions may be under- or over-represented depending on the instrument hierarchy of the originating trial.

### 2.5. Limitations of the Methodology

A narrative selection necessarily reflects the authors’ interpretation and may underweight literature outside the selection. The pipeline section relies partly on conference data not yet peer-reviewed; these are marked. Reference [14] has a PMC identifier but the formal DOI is pending capture at submission. The proposed selection process (Section 6) is conceptual and has not been prospectively validated.

## 3. Pathophysiology, Endotypes, and Predictive Biomarkers

### 3.1. The Mast Cell as the Biological Foundation

CSU is an immunologically driven disease characterised by cutaneous mast-cell activation. The mast cell is the biological substrate on which the targeted therapies discussed in this review act; the molecular detail of its activation underpins the clinical reasoning developed in the selection process. The principal effector pathway is engagement of FcεRI by IgE cross-linked by an external allergen, an IgE autoantibody against a self-antigen (type I autoallergic CSU), or an IgG autoantibody against IgE or against FcεRI itself (type IIb autoimmune CSU) [15,16]. FcεRI aggregation triggers a tyrosine-kinase cascade in which Lyn, Syk and Bruton tyrosine kinase (BTK) are central; downstream activation of phospholipase Cγ mobilises intracellular calcium and culminates in degranulation. Histamine, tryptase, leukotriene C4 and a battery of cytokines are released, driving wheal formation, angioedema and pruritus. The same intracellular machinery is engaged by parallel activating receptors. The most clinically relevant in chronic urticaria are MRGPRX2—activated by substance P and by certain drugs—and the complement receptor C5aR1 [15]. KIT, the receptor for stem-cell factor, does not transmit acute degranulation signals; it supports skin-mast-cell survival, and its blockade results in mast-cell depletion rather than activation inhibition [9].

### 3.2. Two Endotypes That Organise the Heterogeneity

Two clinical endotypes have been consolidated in recent reviews [15,16,17]. Type I autoallergic CSU is characterised by IgE autoantibodies against self-antigens (thyroid peroxidase, IL-24 and others), comparatively higher total serum IgE, and a relatively rapid response to omalizumab. Type IIb autoimmune CSU is defined, under strict triple-positivity criteria (positive autologous serum skin test plus IgG autoantibody immunoassay plus basophil activation test), by IgG autoantibodies against IgE or FcεRI, lower total IgE, basopenia, eosinopenia, higher prevalence of concomitant autoimmune disease, and partial or delayed response to omalizumab [15]. This strictly defined subgroup accounts for fewer than 10% of cases [15,16]; in practice, the distinction from type I is often blurred, and some patients display features of both. Gain-of-function variants in MRGPRX2 have been associated with higher disease activity and suggest that the MRGPRX2 axis may constitute a third endotype not yet operationalised in routine practice [18].

### 3.3. Mapping Endotype to Mechanism

Each therapeutic axis acts on a different node of the cascade, with implications for which endotype is most likely to benefit (Figure 1). Anti-IgE neutralisation acts upstream of FcεRI aggregation by depleting free IgE and inducing FcεRI down-regulation; it is mechanistically aligned with type I CSU and partially active in type IIb, where IgE autoantibody concentrations are lower. Anti-IL-4Rα receptor blockade acts on the type 2 cytokine environment, lowering IL-4- and IL-13-driven IgE class-switching and mast-cell priming; its rationale is strongest where type 2 inflammation is sustaining the disease. Anti-KIT mast-cell depletion acts at the level of effector-cell survival, removing the source of degranulation regardless of which activating receptor is engaged. Oral BTK inhibition acts at the intracellular convergence of FcεRI signalling and attenuates activation independently of IgE concentration.

Taken together, these four axes do not address a single rate-limiting step; they address four parallel rate-limiting steps operating at different layers of the pathophysiology. Each mechanism therefore has a distinct zone of biological plausibility, determined by which step is rate-limiting in the individual patient—the observation that anchors the predictive-factor reasoning developed below. Section 4 appraises each mechanism, Section 5 compares them, and Section 6 integrates the evidence into a selection process.

Predictive biomarkers (Figure S1). Baseline markers help anticipate response and are most useful when read by mechanisms. For omalizumab, a normal-to-high total IgE and elevated basophil FcεRI expression predict a faster and more complete response, whereas low total IgE (typically < 40–50 IU/mL), IgG-anti-thyroid-peroxidase positivity and a type IIb autoimmune profile predict a slower or incomplete response [14,15,19]. Remibrutinib and barzolvolimab act independently of IgE concentration, so IgE-based markers do not predict their effect; comorbidity profile (type 2 versus autoimmune) remains the most practical proxy when basophil-based assays (BAT, BHRA) are unavailable [17,19]. These directional associations are summarised by agent in Table S3; none is yet validated for routine treatment selection.

The intracellular cascade proceeds from FcεRI aggregation through Lyn, Syk and Bruton tyrosine kinase (BTK) to phospholipase Cγ, raising intracellular calcium and triggering degranulation; each agent acts at a distinct node—omalizumab upstream of FcεRI, dupilumab on the parallel IL-4Rα axis, remibrutinib at BTK, and barzolvolimab at KIT-dependent mast-cell survival.

## 4. Drug-by-Drug Evaluation and Critical Appraisal

For each agent we report trial data, regulatory approvals, a critical appraisal, the interpretation we draw, and open questions. The pivotal phase 3 trials are summarised in Table S2. The critical appraisal is the principal value-added over descriptive review structures and includes concrete methodological caveats.

### 4.1. Omalizumab—Anti-IgE Neutralisation

**Trial data (Level 1 + 2).** Pivotal efficacy was established in ASTERIA I, ASTERIA II and GLACIAL, with meaningful effects within 4 to 12 weeks [20,21]. The 2025 network meta-analysis by Xiong and colleagues—15 randomised trials, 4913 patients—ranked omalizumab 300 mg every four weeks ahead of dupilumab and remibrutinib for disease control and symptom remission at weeks 12 and 24 [13].

**Approvals.** Omalizumab has been approved for CSU in adults and adolescents (≥12 years) since 2014 in Europe and the United States. The first omalizumab biosimilar (CT-P39) was approved by the EMA in March 2024 and by the FDA in March 2025 [4].

**Critical appraisal.** The pivotal trials excluded the most severely refractory tail (e.g., patients on omalizumab in earlier studies, patients with active angioedema requiring rescue therapy). Outcome definitions in ASTERIA and GLACIAL predate the 2014 standardisation of UAS7 cut-points used in current practice, which complicates effect-size comparison with post-2020 trials. The trial populations were predominantly North American and European; East Asian, South Asian, Latin American and African populations are under-represented in the pivotal cohorts and remain so in subsequent omalizumab real-world data. Sensitivity analyses in Xiong and colleagues’ network meta-analysis are not stratified by trial generation, and the omalizumab-favouring ranking partly reflects the cumulative weight of pre-2020 trials with comparatively higher placebo responses—a methodological point that deserves explicit acknowledgement.

**Interpretation.** Omalizumab appears to remain the most appropriate first-line antibody on the basis of guideline-level recommendation, depth of clinical experience and the recent quantitative ranking [2,13]. In partial responders at six months, dose escalation to 600 mg every four weeks or shortening of the dosing interval may be reasonable before considering a mechanism switch; real-world evidence supports updosing or interval shortening before a mechanism switch, with a good safety profile, although practice varies by jurisdiction (Level 4) [22].

**Positioning.** Omalizumab is the reference against which all second-line options are evaluated; mechanism-guided alternatives become relevant once omalizumab at full dose has not achieved disease control. The next sections examine those alternatives.

**Open questions.** Predictors of late response and of relapse after withdrawal are incompletely characterised. The clinical effect of biosimilar substitution on real-world persistence has not been reported.

### 4.2. Dupilumab—Anti-IL-4Rα Receptor Blockade

**Trial data (Level 1 + 4).** The LIBERTY-CSU CUPID programme comprised CUPID-A (omalizumab-naive; positive primary endpoint), CUPID-B (omalizumab-refractory; negative primary endpoint) and CUPID-C (confirmatory naive; positive) [3,5]. Initial real-world data on transitioning patients from omalizumab to dupilumab were reported by Hayama and colleagues in 2025: the mean Urticaria Control Test score rose from 6.5 ± 2.2 at dupilumab initiation to 8.4 ± 3.0 at 1 month (*p* = 0.016) and 8.1 ± 3.9 at 4 months (*p* = 0.045), although individual responses were heterogeneous [23] (Level 4). In Xiong and colleagues’ meta-analysis, dupilumab provided sustained itch relief with slower onset of efficacy [13].

**Approvals.** Dupilumab was approved for CSU in Japan in February 2024 and in the United States in April 2025 in patients ≥12 years symptomatic on sgH1-AH; the US indication was extended to children aged 2–11 years in April 2026. The United States label is anchored on CUPID-A and CUPID-C in omalizumab-naive patients [4].

**Critical appraisal.** Three concrete caveats apply. First, the LIBERTY-CSU CUPID-B trial had a randomised horizon of 24 weeks; dupilumab’s effects accrue over weeks, and longer follow-up might have detected delayed benefit, particularly in patients with sustained type 2 inflammation. Second, the eligibility criteria for CUPID-B (incomplete response to or intolerance of omalizumab) were probably enriched for non-type-2 endotypes precisely because partial responders with type 2 comorbidity tend to be continued on omalizumab in routine practice rather than enrolled in alternative-mechanism trials. Third, the Hayama 2025 transition cohort is small (*n* = 12), single-centre, and Japanese; the favourable signal it suggests should not be over-read [23]. The CUPID-A vs. CUPID-B asymmetry is, on present evidence, more consistent with selection effects than with a definitive verdict that IL-4Rα blockade does not work in omalizumab-refractory CSU. This interpretation is testable: an endotype-enriched phase 3 trial in CUPID-B-like populations stratified by type 2 markers would settle the question.

**Interpretation.** Dupilumab is a rational option where type 2 comorbidity coexists, but its Level 1 evidence is confined to omalizumab-naive patients (CUPID A/C); in omalizumab-refractory disease the dedicated trial (CUPID B) failed its primary endpoint and was stopped for futility, so this positioning remains unsupported rather than merely under-studied.

**Positioning.** Dupilumab is best supported when type 2 comorbidity is present; in the strictly omalizumab-refractory non-type-2 patient it occupies a lower-evidence position. The CUPID-A-versus-CUPID-B contrast—a positive readout in omalizumab-naive patients and a negative readout in omalizumab-refractory patients—is, on present evidence, more consistent with selection effects than with mechanism failure, and the endotype-enriched trial proposed in Section 7 would test this directly.

**Open questions.** Whether endotype enrichment recovers a positive CUPID-B-like population remains untested prospectively. Paediatric CSU data are limited.

### 4.3. Remibrutinib—Oral BTK Inhibition

**Trial data (Level 1 + 2 + 4).** REMIX-1 and REMIX-2 (NEJM, March 2025) enrolled approximately 925 adults with sgH1-AH-refractory CSU and reported significant UAS7 reductions versus placebo, with onset by week 1 [6]. Well-controlled disease (UAS7 ≤ 6) at week 12 was achieved by 49.8% versus 24.8% (REMIX-1) and 46.8% versus 19.6% (REMIX-2) [4]. A subsequent systematic review and meta-analysis (three RCTs, *n* = 997; two single-arm studies, *n* = 280) reported a mean UAS7 difference of −7.81 (95% CI −10.29 to −5.33) and sustained efficacy through 24 to 52 weeks [24]. The Japanese open-label BISCUIT cohort (*n* = 71) reported that 53.6% of patients reached UAS7 ≤ 6 and 30.4% reached UAS7 = 0 at week 52 [25].

**Approvals.** FDA approval was granted on 30 September 2025 (marketed as Rhapsido) for adults with sgH1-AH-refractory CSU [6], with no paediatric indication. EMA authorisation should be verified at submission.

**Critical appraisal.** Two concerns deserve emphasis. First, the REMIX programme had a recruitment imbalance toward Central and Eastern European sites, which affects external validity for North American and East Asian practice. Second, the BISCUIT extension is single-arm, open-label and Japanese-only (*n* = 71); its 52-week data are reassuring but cannot be generalised across populations or used as a quasi-randomised durability source. Third, although the short-term safety profile is acceptable, the long-term safety of BTK inhibition in non-oncological populations remains immature beyond 52 weeks, particularly for cardiovascular, hepatic and infectious outcomes [26]. Xiong and colleagues’ meta-analysis identified remibrutinib as the agent with the greatest Dermatology Life Quality Index improvement and the second-greatest UAS7 reduction across the three approved options [13]—a reminder that the apparent ranking depends on the endpoint selected.

**Interpretation.** Remibrutinib offers an oral alternative with rapid onset and an acceptable short-term safety profile. As an IgE-independent mechanism, BTK inhibition is mechanistically plausible in type IIb autoimmune CSU and in omalizumab-refractory patients, but its preferability over a switch in antibody mechanism in any specific subgroup is not directly addressed by the available trials.

**Positioning.** Remibrutinib is a rational option after omalizumab because REMIX is the only pivotal phase 3 programme to date that included omalizumab-experienced patients in its primary analysis [6]: engaging a parallel intracellular mechanism after the upstream IgE block produced additional benefit. The ligelizumab failure [8] is consistent with the converse—that deepening the same upstream IgE block adds little. These observations inform the selection reasoning developed in Section 6.

**Open questions.** Long-term safety beyond 52 weeks in non-oncological use, and real-world durability across populations, will become clearer with the prospective registries now starting (NCT07358780, NCT07358364, NCT07408219).

### 4.4. Barzolvolimab—Anti-KIT Mast-Cell Depletion (Investigational)

**Trial data (Level 3).** Barzolvolimab is a humanised anti-KIT monoclonal antibody that depletes skin mast cells by blocking stem-cell-factor-dependent KIT signalling—an approach distinct from the three approved agents in that it removes the effector cell rather than inhibiting its activation. In a phase 1b multiple-ascending-dose trial (NCT04538794), rapid symptom reduction within one week was sustained over 12 weeks, with 71% of treated patients reaching well-controlled disease (UAS7 ≤ 6) and 57% complete response (UAS7 = 0); the kinetics paralleled serum tryptase suppression, consistent with mast-cell depletion, and patients with and without prior omalizumab responded similarly [27]. The phase 2 dose-finding trial in antihistamine-refractory CSU (NCT05368285; *n* = 207) randomised patients to barzolvolimab 75 mg every 4 weeks, 150 mg every 4 weeks, 300 mg every 8 weeks, or placebo; the placebo-adjusted change in UAS7 at week 12 was −12.55 (95% CI −16.56 to −8.55) for 150 mg every 4 weeks and −13.41 (−17.47 to −9.34) for 300 mg every 8 weeks (both *p* < 0.0001), with complete response (UAS7 = 0) in 51.1% and 37.5% versus 6.4% for placebo, and responses again similar irrespective of prior omalizumab exposure [28].

**Regulatory status.** Barzolvolimab is investigational and is not approved for CSU in any jurisdiction. Two phase 3 trials are underway—EMBARQ-CSU1 (NCT06445023) and EMBARQ-CSU2 (NCT06455202)—enrolling biologic-naive and biologic-experienced adults, with a pre-specified omalizumab-refractory subgroup analysis and a treatment-free period; primary completion is expected in October 2026.

**Critical appraisal.** The evidence is phase 2 and therefore hypothesis-generating for clinical positioning. Three caveats apply. First, efficacy was shown over 12 weeks; durability and the trajectory of mast-cell repopulation after withdrawal are unknown and are a defining question of the phase 3 programme. Second, the consistent response regardless of baseline omalizumab exposure is mechanistically attractive but has not yet been confirmed in an adequately powered omalizumab-refractory population. Third, mast-cell depletion is a more profound intervention than receptor-level inhibition, so the consequences of sustained KIT blockade require longer-term phase 3 safety data.

**Safety (Level 3).** Across the phase 1b and phase 2 trials, barzolvolimab was generally well tolerated; the characteristic, mechanism-related adverse events are changes in hair colour (reported in approximately 14% of treated patients), reversible decreases in neutrophil count (approximately 9%) and skin hypopigmentation (approximately 1%), reflecting KIT expression in melanocytes and neutrophil precursors [11,27,28]. No safety signal beyond these mechanism-linked effects has been established, but the safety horizon remains short.

**Interpretation.** Barzolvolimab is the most advanced mast-cell-depleting strategy in CSU and is mechanistically rational where activation-level blockade is insufficient—for example, in IgE-low type IIb autoimmune disease and in omalizumab-refractory patients—because it acts on effector-cell number rather than on a single activating receptor (Level 3, hypothesis-generating). Its position relative to the approved agents cannot be defined until the EMBARQ-CSU readouts; it is therefore discussed here as an investigational option, not as a current recommendation.

**Open questions.** Whether mast-cell depletion produces durable remission after withdrawal, whether the hair-colour and neutrophil effects remain acceptable over longer exposure, and whether anti-KIT therapy is preferentially effective in molecularly defined subgroups are the principal unknowns awaiting the phase 3 data.

### 4.5. Beyond Symptom Suppression: Is Disease Modification Plausible?

The pivotal trials of all three approved mechanisms in CSU are designed to demonstrate symptom suppression on treatment, not disease modification beyond it. Whether any of these agents can produce durable remission after withdrawal—and whether they can shift the natural history of the underlying disease process—is a question the field is only beginning to ask.

For omalizumab, observational data and small withdrawal protocols suggest that a meaningful proportion of patients remain in remission for several months after discontinuation, though most relapse within a year; predictors of stable post-treatment remission are not well established. In a real-world cohort of 102 patients, omalizumab could be discontinued in two-thirds of complete responders using gradually lengthened dosing intervals, with 58% still in remission and 42% relapsing at a mean of 12 months; relapses re-responded to re-treatment [29]. For dupilumab, the effect on IgE production is well characterised in atopic dermatitis but its translation to CSU disease modification is unknown; the slow onset of effect in CUPID-A is consistent with a class-switching-modifying mechanism that might require longer exposure to produce durable change. For remibrutinib, the irreversibility of covalent BTK binding raises the possibility that intermittent rather than continuous dosing might be feasible; this has not been tested in CSU.

For anti-KIT depletion, the question is qualitatively different. If skin mast cells are depleted during treatment, repopulation after withdrawal depends on stem-cell-factor-driven recruitment and on whether the repopulating pool carries the disease memory or arises de novo. Phase 2 data suggest sustained benefit during treatment; the post-withdrawal trajectory will be a critical readout of the EMBARQ-CSU programme and a defining test of whether mast-cell depletion is a maintenance therapy or a potentially curative one.

Taken together, the disease-modification question is unresolved across the four mechanism classes, and the design of withdrawal sub-studies in real-world registries is the most efficient path to answering it.

### 4.6. Section Synthesis

Across the four approved or near-approved mechanisms, the available evidence supports several integrative observations. The consistency of omalizumab’s effect across ASTERIA, GLACIAL, twelve years of real-world cohorts and the recent network meta-analysis is the most robust pattern in the field. The CUPID-A/CUPID-B asymmetry is the most informative single result of the last two years and is more readily explained by selection than by mechanism failure, although the matter is not settled. The REMIX consistency across two trials and the rapid onset are well documented but the safety horizon remains short. Anti-KIT depletion has phase 2 signals strong enough to motivate two phase 3 trials but is not yet decision-relevant in routine practice. The integrative observation is that none of these mechanisms is universally preferable; each has a distinct zone of biological plausibility, which the comparative analysis (Section 5) and the selection process (Section 6) make explicit.

## 5. Comparative Analysis

This section weighs the four agents against one another(Table S1): first through the only published quantitative indirect comparison, then through the most clinically informative pairwise considerations. Because no head-to-head data yet exist for most pairs, the comparison is necessarily interpretive.

### 5.1. Critical Engagement with Xiong and Colleagues’ Network Meta-Analysis

Xiong and colleagues’ network meta-analysis [13] is the single most consequential indirect comparison published in this field to date and merits sustained engagement rather than narrative citation. Fifteen RCTs and 4913 patients were pooled; omalizumab 300 mg ranked first on disease control (SUCRA values were highest), dupilumab third on the same endpoint and second on sustained itch relief, and remibrutinib first on DLQI improvement and second on UAS7 reduction. Several caveats temper the interpretation.

First, the heterogeneity across pooled trials is non-trivial. ASTERIA (2013) and GLACIAL (2013) recruited under endpoint conventions that pre-date the 2014 UAS7 standardisation and used predominantly North American and European sites with relatively high placebo responses; CUPID (2024) and REMIX (2025) were conducted under contemporary standards with different placebo dynamics. Without trial-generation stratification, the omalizumab-favouring ranking partly reflects accumulated evidence from a different methodological era.

Second, the certainty of the estimates is variable. For omalizumab 300 mg, the certainty of the disease-control estimate is high; for dupilumab in omalizumab-refractory populations, the certainty is low because CUPID-B contributes negative data with substantial precision concerns at week 24; for remibrutinib, the certainty for short-term outcomes is high but for sustained 24-week effects relies in part on extension data outside the randomised window.

Third, the meta-analysis cannot capture the subgroup effects most relevant to the second-line decision. The omalizumab-naive-versus-omalizumab-refractory dimension on which CUPID-A and CUPID-B differ is precisely the dimension that matters for agent selection, and a meta-analysis pooled across populations cannot resolve it.

The integrative reading is that Xiong and colleagues’ ranking is consistent with first-line use of omalizumab but does not adjudicate the second-line decision; quantitative comparison at the subgroup level is the work that remains to be done.

A larger network meta-analysis underpinning the 2025 AAAAI/ACAAI Joint Task Force guideline update (93 studies, 42 interventions) reached a concordant conclusion: with high certainty, standard-dose omalizumab (300 mg every 4 weeks) and remibrutinib were among the most effective options across multiple patient-important outcomes; dupilumab improved urticaria activity, with uncertain effects on quality of life and angioedema; and cyclosporine was effective but among the least safe [30]. This independent synthesis reinforces—rather than contradicts—the first-line positioning of omalizumab adopted here, while neither network meta-analysis provides the operational, patient-level selection process this review proposes.

### 5.2. Comparative Considerations Across Mechanism Pairs

This subsection addresses the most clinically informative pairwise comparisons.

**Omalizumab versus dupilumab.** Both subcutaneous antibodies act on adjacent but distinct nodes (free IgE versus IL-4/IL-13 signalling). Pooled disease control favours omalizumab [13]. Endotype responsiveness differs: omalizumab is best aligned with type I autoallergic CSU, and dupilumab with type-2-driven disease and concomitant type 2 comorbidity. The principal limitation of this comparison is the absence of any head-to-head trial.

**Dupilumab versus remibrutinib.** The two newest mechanisms will be compared head-to-head in RECLAIM (NCT06868212, primary completion February 2027) [10]. The trial’s week-4 primary endpoint is designed to capture the most discriminating dimension: onset of action. Remibrutinib achieves meaningful UAS7 reductions by week 1 in REMIX [6]; dupilumab’s onset is on the order of 12 weeks in CUPID-A and CUPID-C [3,5]. Beyond onset, the differentiators are: population (remibrutinib adults only; dupilumab ≥2 years); administration (oral versus subcutaneous); comorbidity profile; endotype rationale; safety horizon. The principal limitation is that RECLAIM is incomplete.

**Anti-IgE-versus-anti-KIT strategies.** This involves a comparison between mature and investigational. Anti-IgE neutralisation prevents activation; anti-KIT depletion removes the effector. Phase 2 data on barzolvolimab support a favourable profile in patients refractory to omalizumab [9]. Whether depletion translates into durable benefit and whether post-withdrawal mast-cell repopulation will be problematic remain to be determined by the EMBARQ-CSU readouts.

**Figure 2:**
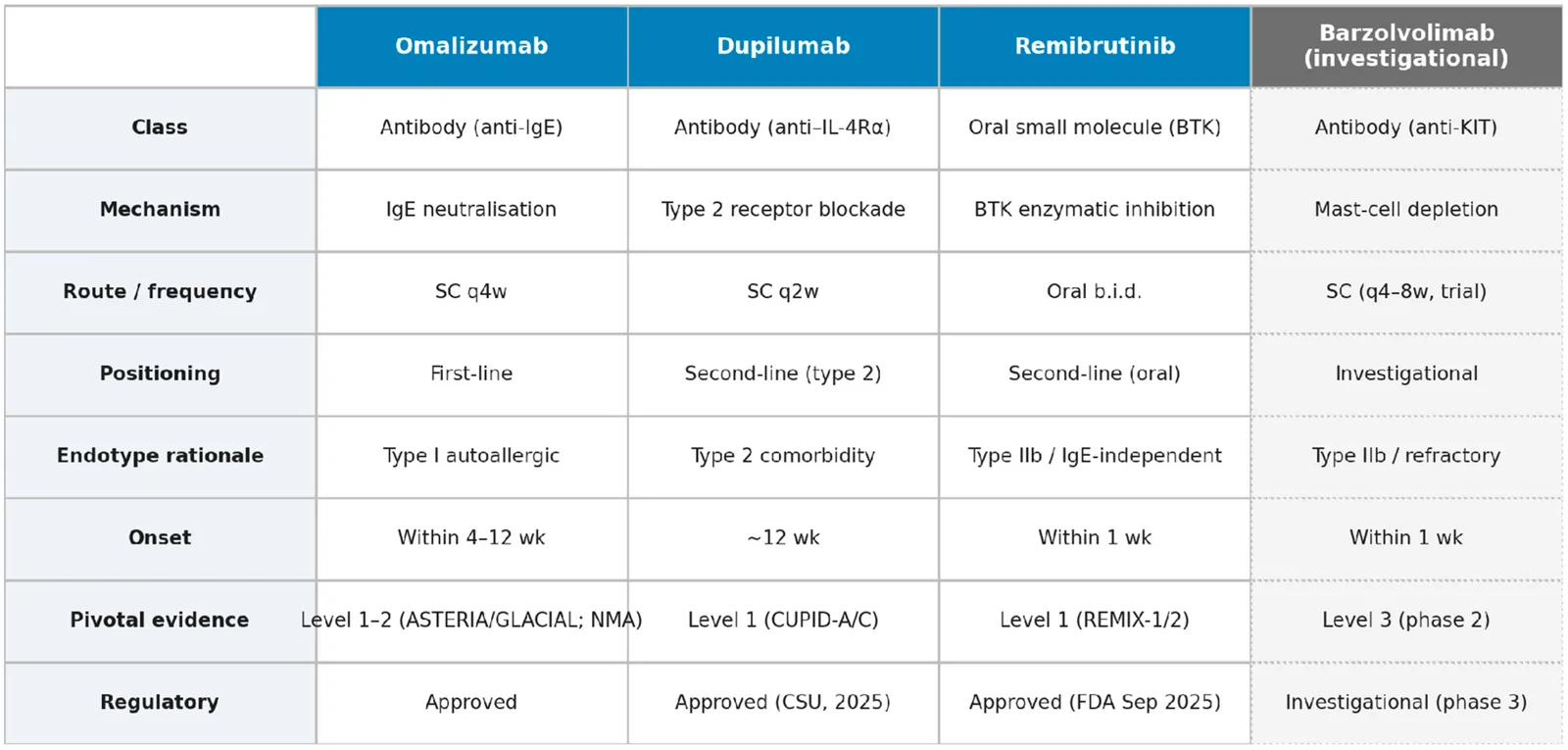
Comparative profile of targeted therapies for chronic spontaneous urticaria (CSU). Approved agents (omalizumab, dupilumab, remibrutinib) and the investigational anti-KIT antibody barzolvolimab are compared across drug class, molecular mechanism, route and dosing frequency, current positioning in the treatment algorithm, endotype rationale, expected time to onset of response, level of pivotal evidence and regulatory status. Levels of evidence are graded 1 (randomised controlled trials/meta-analyses) to 3 (phase 2 data). SC, subcutaneous; q2w/q4w/q4–8w, every 2/4/4–8 weeks; b.i.d., twice daily; IgE, immunoglobulin E; IL-4Rα, interleukin-4 receptor alpha; BTK, Bruton’s tyrosine kinase; KIT, stem-cell-factor receptor; NMA, network meta-analysis.

**Antibody therapy versus oral BTK inhibition.** This is not solely a pharmacological question. Patient preference for oral therapy, when efficacy and safety are broadly comparable, is a legitimate dimension. The asymmetry in long-term safety is the principal point of disclosure: anti-IgE has >10 years of real-world experience; anti-IL-4Rα blockade has accumulated experience across indications since 2017; oral BTK inhibition outside oncology has a 52-week horizon in CSU and a smaller cumulative non-oncological dataset [26] (Figure 2, Box 2).

Box 2 **Live controversies in the field**

  (i) Is CUPID-B a verdict on dupilumab in refractory CSU, or a selection artefact? The mechanism-limited and selection interpretations remain unresolved and are testable by endotype-enriched re-trial. (ii) Should remibrutinib be considered first-line in type IIb autoimmune CSU? The mechanistic rationale is strong but no randomised evidence directly supports it; the position is explicitly a proposal and hypothesis-generating. (iii) Will anti-IgE biosimilars displace remibrutinib on cost-per-responder grounds before remibrutinib accumulates its long-term safety record? This is a health-economic rather than a clinical question and will be settled by HTA bodies. (iv) Should mechanism-combining strategies (omalizumab + remibrutinib) be tested in highly refractory disease? The current answer is no in routine practice and yes in registry-embedded research.

## 6. An Approach to Drug Selection

### 6.1. Relationship to the Guideline

The 2022 EAACI/GA^2^LEN/EuroGuiDerm/APAAACI guideline remains the reference standard and is not superseded here [2]. The selection process and the proposed repositioning set out below operate beyond the guideline ladder: they address the second-line choice among mechanistically distinct agents that the guideline does not yet adjudicate. Every position is annotated with its level of evidence and is presented as a proposal and as hypothesis-generating, not as established or guideline-endorsed practice.

### 6.2. First-Line Position

Omalizumab appears to remain a reasonable first-line antibody in most patients with sgH1-AH-refractory CSU, based on guideline-level recommendation [2]; the recent network meta-analysis ranking [13]; the depth of evidence, including pregnancy [31] and paediatric data (Table S6); and over a decade of real-world experience. Omalizumab is therefore positioned as first-line in both endotypes (type I and type IIb), with the caveat that response in type IIb may be slower and less complete.

### 6.3. Defining Non-Response (Level G–6)

Omalizumab non-response: UAS7 reduction <50% from baseline or UAS7 > 6 after six months at 300 mg every four weeks. Dose escalation to 600 mg every four weeks or interval shortening may be considered, supported by real-world evidence (Level 4) [22]; local label permissibility should be verified. Remibrutinib non-response: UAS7 reduction <50% or UAS7 > 6 at week 4–12. Dupilumab non-response: UAS7 reduction <50% or UAS7 > 6 at week 12–16.

### 6.4. Predictive Factors and Patient-Tailored Second-Line Choice

Second-line selection is oriented by predictive factors (Table S3) rather than applied uniformly: IgE-based markers and the comorbidity profile guide the choice, recognising that low total IgE, type IIb autoimmunity and omalizumab-experienced status favour IgE-independent mechanisms, whereas type 2 comorbidity favours anti-IL-4Rα blockade [14,19]. Because basophil-based assays are research-grade, comorbidity profile is the most practical proxy in routine care.

A pragmatic surrogate for type IIb autoimmune CSU (proposal). Because basophil activation and histamine-release assays remain research-grade, we propose—as a hypothesis-generating aid—a pragmatic composite to flag likely type IIb autoimmune disease in routine care: low total IgE (typically < 40–50 IU/mL), basopenia, anti-thyroid-peroxidase positivity or another autoimmune comorbidity, and a slow or incomplete response to omalizumab. A patient meeting several of these criteria may be prioritised for an IgE-independent mechanism; the composite requires prospective validation before any routine use [14,15,17,19].

After omalizumab non-response, the choice between remibrutinib, dupilumab and (in due course) anti-KIT depletion depends on residual driver, comorbidity profile and patient preference. Remibrutinib is the most evidence-supported option in type IIb autoimmune CSU and in omalizumab-experienced patients without type 2 comorbidity (Level 1). Dupilumab (anti-IL-4Rα) is a rational option in patients with concomitant type 2 disease, atopic dermatitis or asthma, in whom a single biologic may address both conditions. Its Level 1 evidence, however, derives exclusively from omalizumab-naive patients (CUPID Studies A and C), in whom the drug was evaluated as an alternative to omalizumab rather than as a successor to it. In omalizumab-intolerant or incomplete responders, the dedicated Study B did not meet its primary endpoint and was terminated for futility at prespecified interim analysis, with only marginal effects on itch and urticaria activity. Use after omalizumab failure is therefore not supported by the pivotal programme and currently rests on mechanistic plausibility and uncontrolled reports. Cyclosporine retains a role as a later option in patients refractory to multiple targeted therapies, with contraindications or in resource-limited settings.

After dupilumab non-response, switching to remibrutinib is reasonable in most patients; barzolvolimab may enter the decision tree pending phase 3 readout. Combination therapy across mechanisms is not currently supported by randomised evidence (Level 6).

After remibrutinib non-response, switching to anti-IgE or anti-IL-4Rα may be considered according to endotype and comorbidity profile.

### 6.5. Decision Algorithm

**Figure 3:**
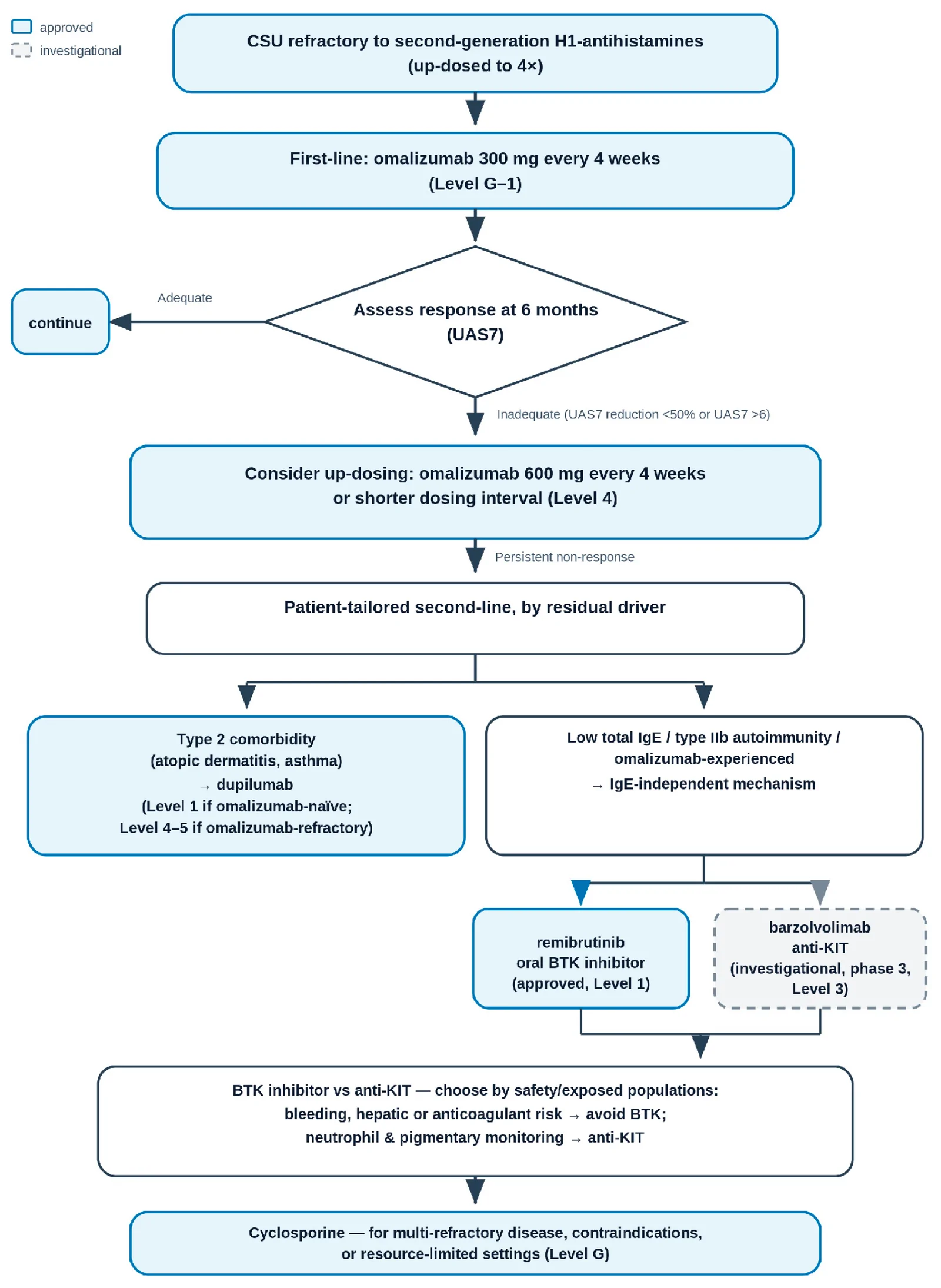
A patient-tailored approach to targeted therapy in antihistamine-refractory chronic spontaneous urticaria. Regulatory status is colour-coded (approved, solid; investigational, grey dashed). The algorithm is hypothesis-generating and is not a clinical guideline.

The selection process can be summarised as a stepwise algorithm (Figure 3). (1) Confirm sgH1-AH-refractory CSU after up-dosing antihistamines to fourfold. (2) Start omalizumab 300 mg every 4 weeks as first-line targeted therapy (Level G–1) [2]. (3) Assess response at six months using UAS7. (4) If the response is adequate, continue; if inadequate (UAS7 reduction < 50% or UAS7 > 6), consider up-dosing omalizumab to 600 mg every 4 weeks or shortening the dosing interval, supported by real-world evidence (Level 4) [22]. (5) In persistent non-response, choose a patient-tailored second-line option by residual driver: type 2 comorbidity (atopic dermatitis, asthma) favours dupilumab (Level 1 in omalizumab-naive CSU; Level 4–5 in omalizumab-refractory disease) [3,5]; low total IgE, type IIb autoimmunity or omalizumab-experienced status favours an IgE-independent mechanism—remibrutinib now (Level 1) [6], with anti-KIT depletion (barzolvolimab) an investigational future option (Level 3) [28]. (6) When choosing between BTK inhibition and anti-KIT depletion, weigh safety and exposed populations: bleeding, hepatic or anticoagulant risk argues against BTK inhibition, whereas neutrophil and pigmentary monitoring are the relevant considerations for anti-KIT therapy (Table S5) [11]. (7) Reserve cyclosporine for disease refractory to multiple targeted therapies, for contraindications, or for resource-limited settings (Level G) [2]. The regulatory status of every branch is labelled; investigational options are not current recommendations.

### 6.6. Proposed Repositioning in Clinical Practice

Beyond the guideline ladder we propose three evidence-graded repositionings, each a hypothesis-generating proposal rather than established practice. First, in omalizumab-naive patients with prominent type 2 comorbidity, dupilumab may reasonably be brought forward as an early antibody choice—for the dual benefit on CSU and the atopic comorbidity—rather than reserved strictly to later lines (Level 1 in naive CSU; proposal for sequencing) [3,5]. Second, after omalizumab failure, an oral IgE-independent mechanism (remibrutinib) is proposed as the preferred next step in type IIb autoimmune or omalizumab-experienced disease, on the strength of its inclusion of omalizumab-experienced patients in the pivotal programme (Level 1) [6]. Third, anti-KIT mast-cell depletion (barzolvolimab) is positioned as the most plausible future option for disease refractory to receptor-level inhibition, explicitly investigational pending the EMBARQ-CSU readouts (Level 3) [28]. None of these positions is guideline-endorsed; each requires prospective validation, ideally head-to-head (for example, RECLAIM) and in endotype-enriched designs.

## 7. Future Perspectives

The next two to three years will be shaped less by additional single-drug phase 3 trials and more by the integration of mechanistic stratification into clinical decision-making, by registry-embedded pragmatic research, and by trial-design innovations capable of answering questions that conventional phase 3 cannot.

**Precision medicine and molecular endotyping.** The transition from clinical phenotyping (severity, antihistamine response) to molecular endotyping has been argued for in recent reviews [17,32]. The practical obstacle is the limited availability of validated assays: basophil activation testing and basophil histamine-release assays remain research tools outside specialist centres. A reasonable two-year objective is the validation of pragmatic surrogate composites sufficient to guide mechanism selection in routine practice.

**Biomarker-driven sequencing.** Total IgE, anti-thyroid antibodies and basophil counts have modest predictive value individually but may combine into useful composites [17,32,33]. Whether these composites will outperform clinical phenotype alone in head-to-head pragmatic trials is the empirical question.

**Omics, transcriptomics and single-cell sequencing.** Skin mast cells in CSU are heterogeneous at the single-cell level, and the response to systemic therapy may differentially affect subpopulations. Single-cell transcriptomic studies of pre- and post-treatment skin biopsies would establish whether anti-KIT depletion is uniform across subpopulations and whether IL-4Rα blockade reprogrammes type 2 transcriptional signatures. This is the most likely source of the next set of mechanistic insights.

**Artificial intelligence and machine learning.** The CSU treatment-selection problem has the structure typical of indications in which machine-learning approaches may add value: multiple plausible options, heterogeneous responder profiles, and accumulating real-world data. Methodological precedents for drug-survival and treatment-persistence modelling exist across inflammatory dermatology; their CSU-specific application will require larger harmonised registries and longitudinal biomarker capture.

**Digital biomarkers.** Patient-reported outcome capture via mobile applications, wearable-derived sleep metrics correlated with itch, and image-based wheal quantification are all feasible at scale and underexploited in CSU.

**Adaptive and basket trial designs.** Conventional phase 3 architectures are not well suited to questions of sequencing, endotype enrichment, or cross-indication mechanism evaluation. Adaptive designs that drop arms and re-weight allocation, basket trials enrolling across CSU, chronic inducible urticaria and idiopathic mast-cell activation, and platform sequencing trials randomising omalizumab failures across remibrutinib, dupilumab and barzolvolimab with a shared control would address the open questions more efficiently. The methodological precedent exists in oncology; the cultural shift is the obstacle.

**Registry-based research.** The remibrutinib real-world programmes (NCT07358780, NCT07358364, NCT07408219) and analogous registries in adjacent inflammatory dermatology indications illustrate the design that will be needed: prospective, multicentre, with harmonised UAS7, UCT and DLQI capture and biomarker sub-studies attached.

**Personalised sequencing strategies. An evidence-graded selection** process such as the one proposed here is an explicit precursor to the personalised sequencing strategies that biomarker availability will eventually support. This process will require iterative revision as evidence accumulates; the current version reflects the evidence available in 2026.

**Pipeline beyond the four current axes.** The future therapeutic pipeline in CSU is summarised in Table S4 MRGPRX2 antagonists were de-prioritised after the EP-262 programme ended [4], but the observation of MRGPRX2 gain-of-function variants associated with higher disease activity [18] argues that the receptor remains a credible target. Next-generation anti-IgE agents (YH35324 and others) will need to clear the ligelizumab cautionary tale [16,34]. Anti-TSLP therapy did not advance in CSU after INCEPTION [35]. Anti-Siglec-8 and anti-Siglec-6 strategies are early in non-CSU indications.

**Access and equity: the biosimilar inflexion point.** Cost-per-responder calculations published before 2024 are already outdated. The arrival of omalizumab biosimilars will reset payer expectations and probably re-anchor first-line economics in omalizumab’s favour through 2027–2028, with implications for lower- and middle-income country access that have not been adequately considered in the contemporary literature. Whether remibrutinib’s oral convenience will outcompete biosimilar omalizumab on net cost, and whether anti-KIT therapy will face a steeper-than-expected reimbursement curve, are open questions for health-technology-assessment bodies.

**CSU as the proving ground for anti-mast-cell biologics.** Most of the antibody-based strategies discussed here have applications beyond CSU. Mastocytosis, mast-cell activation syndrome, hereditary alpha-tryptasemia and certain food-allergy and asthma subtypes will benefit from the lessons of CSU drug development. The reverse is also true: insights from anti-Siglec strategies in eosinophilic gastrointestinal disease, from KIT inhibitors in mastocytosis and from BTK inhibitors in autoimmune cytopenias will inform CSU pipeline decisions.

**A working hypothesis for 2027–2028.** The four-axis algorithm will be augmented by biomarker-driven first-line allocation in centres with access to molecular endotyping, by anti-KIT therapy as a second- or third-line option pending the EMBARQ-CSU readouts, and by an at-least-partial revision of the international guideline. The EMBARQ-CSU and RECLAIM readouts over the next twelve to twenty-four months will be decisive for the positioning of anti-KIT therapy and for the remibrutinib-versus-dupilumab comparison.

**A broader vision for 2030.** Looking five years ahead, the most plausible trajectory is that CSU management will look qualitatively different from the algorithm of 2026. Molecular endotyping at first presentation—combining IgE autoantibody profiling, BAT or surrogate composites, single-cell skin-mast-cell signatures and a small panel of cytokine marker—will likely become the standard entry point, with patients allocated to their predicted-best mechanism rather than to a sequenced default. The four-mechanism armamentarium will probably expand to five or six (next-generation anti-IgE, MRGPRX2 antagonists, expanded BTK class), and at least one therapy will likely demonstrate durable post-withdrawal remission, shifting the conversation from suppression to disease modification. Adaptive and platform trial architectures will probably replace single-drug phase 3 trials as the standard evidence-generation strategy, and registry-embedded pragmatic studies will close the gap between RCT efficacy and real-world effectiveness. Machine-learning models trained on harmonised registries will translate these data into individualised probability estimates of response per mechanism. By 2030, the present review’s selection process will either have been validated, refined, or replaced by a successor; in any of the three cases the manuscript’s value will lie in having made the underlying claims explicit enough to be tested.

## 8. Limitations of This Review

Several limitations should be acknowledged. First, this is a critical narrative review with structured methodology, not a systematic review; selection bias is possible. Second, no head-to-head randomised data are yet available for most of the agents discussed in their CSU indications. Third, long-term safety data for remibrutinib outside oncology and for dupilumab in CSU are immature beyond 52 weeks. Fourth, parts of the pipeline section rely on conference presentations that have not yet been peer-reviewed. Fifth, the decision aid in Section 6 has not been prospectively validated; its specific positions—particularly the consideration of remibrutinib as an alternative in type IIb autoimmune CSU and the use of comorbidity as a proxy for endotype—rest on mechanistic plausibility and limited real-world signal and are presented as hypothesis-generating. Sixth, regulatory information was current as of 23 June 2026. Seventh, biomarker assays (BAT, BHRA) are not universally available. Eighth, the network meta-analysis cited as comparative evidence [13] has the limitations of indirect comparison discussed in Section 5.1. Ninth, the selection process and the proposed repositioning (Section 6) are the conceptual contributions of this review; they are hypothesis-generating, have not been prospectively validated, and are not guideline-endorsed.

## 9. Conclusions

This review offers a patient-tailored, evidence-graded process for choosing among omalizumab, dupilumab, remibrutinib and the investigational anti-KIT antibody barzolvolimab, together with a proposed repositioning of these agents relative to the current guideline ladder. The pathophysiology (Section 3) provides the biological rationale; the drug-by-drug appraisal (Section 4) grades the evidence for each agent; the comparative analysis (Section 5) weighs the agents against one another; and the selection process (Section 6) integrates endotype, comorbidity, prior biologic exposure and safety into a practical, explicitly hypothesis-generating algorithm.

For the prescribing clinician, omalizumab appears to remain a reasonable first-line antibody in most sgH1-AH-refractory CSU, supported by guideline recommendation, by a recent network meta-analysis ranking and by the depth of real-world experience. The second-line decision is no longer routinely cyclosporine; it is a mechanism-informed choice between dupilumab and remibrutinib, with anti-KIT depletion likely to enter the algorithm within twelve to twenty-four months. The choice depends on residual driver, comorbidity profile and patient preference, and the available evidence does not yet adjudicate it unambiguously.

For the antibody-science reader, the post-2025 evidence supports two propositions with wider relevance: that deepening pathway interruption at the same level (the ligelizumab strategy) is unlikely to differentiate, and that benefit beyond omalizumab depends on engaging a parallel mechanism whose appropriateness is endotype-conditioned. These observations motivate the evidence-graded selection process proposed here, which remains a hypothesis-generating proposal awaiting validation by RECLAIM, EMBARQ-CSU and the upcoming real-world registries.

These conclusions are framed as observations and proposals. The decision aid in Section 6 is intended to support clinical reasoning and prospective evaluation; it is not a guideline. A final literature refresh will be performed within two weeks of submission.

## Data Availability

No new data were created or analysed in this study. Data sharing is not applicable to this article.

## Supplementary Materials

The following supporting information can be downloaded at: https://www.mdpi.com/article/10.3390/antib15050088/s1, Table S1: Comparative profile of the principal agents (May 2026). Table S2: Pivotal phase 3 trials in CSU: a critical comparison. Indirect comparison (Xiong and colleagues, network meta-analysis [13]): omalizumab 300 mg ranked first for UAS7, disease control and symptom remission at weeks 12 and 24, and remibrutinib first for DLQI improvement and second for UAS7; no head-to-head trial exists. Table S3: Biomarkers in CSU and their therapeutic implications. Directional associations and evidence levels are consistent with recent predictor reviews [14,15,17,19]. Table S4: Future therapeutic pipeline in CSU. Table S5: Comparative safety and monitoring profile of the targeted agents (underpins the BTK-inhibitor-versus-anti-KIT branch of the algorithm). Table S6: Use of the four agents in special populations. Regulatory ages/status verified at writing; framed cautiously where data are limited. Figure S1: Predictive biomarkers of response by mechanism in CSU. ▲ positive predictor, ▼ negative predictor, ◦ not predictive, —no validated data; the number denotes the highest level of evidence (G, 1–6); * basophil-based assays (BAT, BHRA) are research-grade. Directional associations are consistent with recent predictor reviews [14,15,17,19]; none is yet validated for routine treatment selection.

## Abbreviations

UAS7—Urticaria Activity Score over 7 days, the canonical efficacy endpoint in CSU trials. UCT—Urticaria Control Test. AAS7/AECT—Angioedema Activity Score/Angioedema Control Test. Type I autoallergic CSU—IgE-driven endotype with anti-self IgE autoantibodies. Type IIb autoimmune CSU—IgG-driven endotype with anti-IgE or anti-FcεRI autoantibodies. FcεRI—high-affinity IgE receptor. MRGPRX2—Mas-related G-protein-coupled receptor X2, a neuropeptide-responsive mast-cell receptor. BTK—Bruton tyrosine kinase, intracellular kinase central to FcεRI signalling. BAT—basophil activation test. BHRA—basophil histamine-release assay. Evidence hierarchy—G (guideline) > 1 (phase 3) > 2 (SR/MA) > 3 (phase 2) > 4 (real-world) > 5 (mechanistic review) > 6 (expert/conference).
