# Supplementary material for: Targeted Therapy for Antihistamine-Refractory Chronic Spontaneous Urticaria: A Critical Narrative Review and an Evidence-Graded Approach to Agent Selection
Source: Antibodies (Basel). 2026 Sep 17;15(5):88. doi: 10.3390/antib15050088 (PMC13600177; doi:10.3390/antib15050088)
Supplement: Supplementary file 1 [file antibodies-15-00088-s001.zip › antibodies-4476975-supplementary.pdf]

Tables CSU review

Table S1. — Comparative profile of the principal agents (May 2026).

Feature	Omalizumab	Dupilumab	Remibrutinib	Barzolvolimab (pipeline)
Class	Antibody (anti-IgE)	Antibody (anti-IL-4Rα)	Oral small molecule (BTK)	Antibody (anti-KIT)
Mechanism	IgE neutralisation	Type 2 receptor blockade	BTK enzymatic inhibition	Mast-cell depletion
Route, frequency	SC every 4 weeks	SC every 2 weeks (loading dose)	Oral, 25 mg twice daily	SC every 4–8 weeks (phase 2)
Primary phase 3 publication	Maurer NEJM 2013; Kaplan JACI 2013	Maurer JACI 2024 (CUPID A/B/C)	Metz NEJM 2025 (REMIX-1/2)	EMBARQ-CSU1/2 readout late 2026
Typical onset of meaningful effect	4–12 weeks	~12 weeks (CUPID-A)	~1–2 weeks	~4 weeks (phase 2)
Endotype rationale strongest	Type I autoallergic	Mixed / type 2 comorbid	Type IIb autoimmune (mechanistic)	Type IIb / cellular depletion
Evidence in omalizumab-refractory	n/a (first-line)	CUPID-B NOT met; limited RWE [23]	REMIX populations included omalizumab-experienced	Phase 2 signal incl. omalizumab-pretreated
Approval status (May 2026)	Adults & ≥12 y since 2014; biosimilars 2024–25	CSU: JPN Feb 2024; US Apr 2025	CSU: US Sept 2025, adults only	Phase 3 active, readout late 2026
Safety signals of interest	Anaphylaxis (rare), injection-site	Conjunctivitis, eosinophilia	URI, petechiae; CV/hepatic monitoring	Hair colour changes, neutropenia
Highest level of evidence (first-line)	G	1	1	3

Table S2. — Pivotal phase 3 trials in CSU: a critical comparison. Indirect comparison (Xiong and colleagues, network meta-analysis [13]): omalizumab 300 mg ranked first for UAS7, disease control and symptom remission at weeks 12 and 24, and remibrutinib first for DLQI improvement and second for UAS7; no head-to-head trial exists.

Trial	Drug	Year	N	Population key feature	Primary endpoint	Met?	Methodological strength	Methodological caveat	External validity
ASTERIA I	omalizumab	2013	318	sgH1-AH-refractory	UAS7 change wk 12	Yes	first phase 3 in CSU	endpoint pre-dates 2014 UAS7 standardisation	NA / EU-weighted
ASTERIA II	omalizumab	2013	323	sgH1-AH-refractory	ISS wk 12	Yes	dose-finding component	high placebo response (Maurer NEJM 2013)	NA / EU-weighted
GLACIAL	omalizumab	2013	336	sgH1-AH + H2 + LTRA refractory	ISS wk 12	Yes	most refractory population	endpoint pre-dates UAS7 standardisation	NA / EU-weighted

PEARL-1	ligelizumab	2024	1,072	sgH1-AH-refractory	UAS7 change wk 12	Not superior to omalizumab	active-controlled vs omalizumab	omalizumab arm at ceiling	Global
PEARL-2	ligelizumab	2024	1,078	sgH1-AH-refractory	UAS7 change wk 12	Not superior to omalizumab	replicate of PEARL-1	same caveat	Global
CUPID-A	dupilumab	2024	138	omalizumab-naive	UAS7 change wk 24	Yes	naive population	small sample for phase 3	International
CUPID-B	dupilumab	2024	108	omalizumab-refractory	UAS7 change wk 24	No	distinct refractory population	24-wk horizon may underpower delayed effect; selection may enrich non-type-2	International
CUPID-C	dupilumab	2024	confirmatory	omalizumab-naive	UAS7 change wk 24	Yes	confirmatory replicate	inherited Study A limitations	International
REMIX-1	remibrutinib	2025	~470	sgH1-AH-refractory adults	UAS7 ≤ 6 wk 12	Yes	rapid onset; omalizumab-experienced included	recruitment imbalance Central/Eastern Europe	International
REMIX-2	remibrutinib	2025	~455	sgH1-AH-refractory adults	UAS7 ≤ 6 wk 12	Yes	replicates REMIX-1	same caveat	International
EMBARQ-CSU1	barzolvolimab	ongoing	~963	sgH1-AH-refractory	UAS7 change wk 12	TBD	powered phase 3	results not yet available	Global
EMBARQ-CSU2	barzolvolimab	ongoing	~976	sgH1-AH-refractory	UAS7 change wk 12	TBD	replicate of EMBARQ-CSU1	results not yet available	Global

Table S3. — Biomarkers in CSU and their therapeutic implications. Directional associations and evidence levels are consistent with recent predictor reviews [14, 15, 17, 19].

Biomarker	Availability	Type I marker	Type IIb marker	Predicts omalizumab response	Predicts dupilumab response	Predicts remibrutinib response	Predicts anti-KIT response	Evidence level
Total serum IgE	Universal	↑	↓	Higher IgE → faster, deeper response	Modestly informative	Not established	Not established	2
Anti-TPO IgG	Universal	±	↑	Mixed; some poorer response	Not established	Plausibly favours BTK	Not established	4
Eosinophil count	Universal	Variable	↓	Higher → better response	Higher → better response	Not established	Not established	4
Basophil count	Universal	Variable	↓	Basopenia → poorer/slower	Not established	Not established	Not established	4

ASST	Specialist	±	↑	ASST+ → slower response	Not established	Mechanistically favours BTK	Not established	4
BAT (anti-IgE)	Specialist	Negative	↑	Correlates with IIb response patterns	Not established	Plausibly favours BTK	Not established	3
BHRA	Research	Negative	↑	Correlates with autoimmune CSU	Not established	Plausibly favours BTK	Not established	3
Anti-FcεRI IgG	Research	Absent	↑	Defines type IIb	Not established	Plausibly favours BTK	Plausibly favours depletion	4
MRGPRX2 GOF variants	Research	Not associated	Not associated	Not predictive	Not established	Not established	Plausibly favours depletion	5
Skin mast-cell density	Research	Increased	Increased	Not predictive at baseline	Not established	Not established	Decreases with depletion	5–6
C-reactive protein	Universal	±	±	Higher CRP → poorer in some series	Not established	Not established	Not established	4

Table S4. — Future therapeutic pipeline in CSU.

Drug	Mechanism class	Sponsor	Phase	Trial(s)	Population	Primary endpoint	Expected readout
Barzolvolimab	Anti-KIT antibody	Celldex	3	EMBARQ-CSU1/2	sgH1-AH-refractory	UAS7 wk 12	Oct 2026
Briquilimab	Anti-cKIT antibody	Jasper	1b/2a	BEACON	Refractory	Safety + UAS7	2026
Rilzabrutinib	Reversible BTK inhibitor	Sanofi	2	RILECSU	Refractory	UAS7	2026
YH35324	Next-gen anti-IgE	Yuhan	Early	Various	Adults	Safety + IgE PD	2027–28
RECLAIM head-to-head	Remibrutinib vs dupilumab	Novartis	3b	NCT06868212	sgH1-AH-refractory	UAS7 wk 4	Feb 2027
EP-262	MRGPRX2 antagonist	Escient	Discontinued in CSU	—	—	—	—
Lirentelimab	Anti-Siglec-8	Allakos	Discontinued in CSU	—	—	—	—

Table S5. Comparative safety and monitoring profile of the targeted agents (underpins the BTK-inhibitor-versus-anti-KIT branch of the algorithm).

Feature	Omalizumab	Dupilumab	Remibrutinib	Barzolvolimab
Key adverse events	Injection-site reactions; rare anaphylaxis (class)	Injection-site reactions (~12%); conjunctivitis [11]	Petechiae/bruising (3.8% vs 0.3%, REMIX) [6]; respiratory infections (~11%), headache (~6%); transient liver-enzyme rise [11, 26]	Hair-colour change (~14%); neutropenia (~9%); skin hypopigmentation (~1%) [11, 27, 28]
Routine monitoring	None	None	Liver enzymes; bleeding risk	Neutrophil count
Interactions / cautions	—	—	Caution with anticoagulants/antiplatelets (platelet BTK) [26]	— (investigational)
Safety-data maturity	>10 years real-world	Mature in other type 2 indications	≤52–104 weeks [6, 25]	Phase 2, ≤12 weeks; investigational [28]

Table S6. Use of the four agents in special populations. Regulatory ages/status verified at writing; framed cautiously where data are limited.

Population	Omalizumab	Dupilumab	Remibrutinib	Barzolvolimab
Pregnancy / lactation	Observational data; no clear adverse fetal/maternal signal [31]	Limited data; no clear signal [31]	No data — avoid	No data (investigational)
Paediatric	Approved ≥12 years	≥2 years (FDA, April 2026; ≥12 years since April 2025) [3]	Adults only [6]	Adults; investigational [28]
Elderly	Usable; limited dedicated data	Usable; limited data	Usable; weigh bleeding/hepatic risk	Investigational
Hepatic / bleeding risk	No specific concern	No specific concern	Caution: liver enzymes, bleeding, anticoagulants [26]	Monitor neutrophils [28]
